# Medical and Philosophical Intelligence

**Published:** 1819-06

**Authors:** 


### MEDICAL AND PHILOSOPHICAL INTELLIGENCE.

ALTHOUGH the discoveries which have been made during the late period respecting the pathology of dropsy, clearly indicate the appropriate therapeutical measures in the generality of cases, yet, as it is in physics as well as in morals, maxims and precepts effect but little without examples, we shall adduce some observations of Dr Tweedie,* to shew the efficacy of blood-letting in the dropsical affections consequent on scarlatina.

J. M. æt. five years, was affected with general anasarca, more particularly of the face and upper extremities; frequent short cough, with hurried and oppressed breathing. The urine was scanty; pulse 130, skin hot, and the tongue furred. She had passed through a mild attack of scarlatina about ten days previous to the occurrence of the above symptoms, but had laboured under hooping-cough, in a mild form, for nine weeks. Purgatives of calomel and jalap were exhibited for two days, without benefit. Four ounces of blood were then taken from the arm, which was a little buffy. She experienced immediate relief, and after four days was convalescent.

R. B. æt. five years, eight days after a mild attack of scarlatina, became affected with general anasarca, short dry cough, oppressed breathing, and scanty secretion of urine; pulse 144, and sharp. A "brisk laxative" was given for two days, without any advantage being thence derived; when he was bled to the extent of five ounces, with immediate and decided relief of the pectoral symptoms. The blood was not sizy. Two days afterwards the anasarca was almost gone, and in another day she was declared convalescent.

M. D. æt. fifteen years, about six weeks since laboured under scarlatina. After the disappearance of the efflorescence, she became affected with anasarca, which first appeared on the face, and gradually extended over the trunk and extremities. She complained of pain in the left side of the chest, with dyspnœa and cough, accompanied with a sensation of suffocation when she assumed the horizontal posture. These symptoms had existed eight days, when ten ounces of blood were taken from the arm. The following day she was found to be much relieved; a purgative was then given. The next day, the cough and dyspnœa were nearly gone, and the anasarca much subsided. Four days afterwards she was convalescent.

Dr. Tweedie has witnessed other analogous cases, in which the efficacy of blood-letting was equally prompt and decisive.

* *Edinburgh Medical and Surgical Journal*, No. 58.

In the 233d Number of our Journal, we gave an account of the utility of the *Cubebs*, as a remedy for Gonorrhœa; and we again solicit the attention of medical practitioners to this remedy. Mr. Adams, late of Ceylon, has recently published, in the Edinburgh Medical Journal, some cases shewing its efficacy, and he is disposed to consider it as a specific in the early stage of that disease. Our own experience leads us to think very highly of it, when exhibited before inflammation has come on: at this period, when conjoined with the usual injection of sulphate of zinc, we have but rarely found it to fail in removing the affection within two or three days. After much inflammation has appeared, we have not thought proper to employ it; but Mr. Adams states, that,

"In referring to my case-book, I find those patients who did not derive any benefit from the cubebs, had previously used other remedies for the space of eight or ten days; the inflammatory symptoms had subsided, and the disease had so established itself, as not to be under the control of so simple a remedy."

---

*Observations respecting the Duration of Utero-Gestation and of Incubation, in several Quadrupeds and Domestic Birds.* Extracted from a Memoir read to the *Académie Royale des Sciences* of Paris, by M. Tessier.—In 1,131 *cows*, the shortest period of gestation was 240 days, and the longest, 321 days: difference, 81 days; and, counting from 9 months, 51 days.

In 582 *mares*, which admitted the horse only once, there was, from the shortest gestation (or 287 days) to the longest (419), 132 days; and, reckoning from the 330th day, or eleven months, 89 days.

In 312 *mares*, which had leaped several times, (in dating from the last,) from the shortest gestation, (that is, 290 days,) to 377, the longest, 87 days; and from the 330th, or eleventh month, 47 days of prolongation.

Of two female *asses*, one carried 380 days, or twelve months and twenty days; the other 391 days, or thirteen months and one day.

In 912 *ewes*, the difference was from 146 to 157 days; and from the fifth month to extreme period, seven days.

In eight female *buffaloes*, there was a difference of 27 days between the shortest and longest period; and the latter extended to 338 days, or ten months and 28 days.

Two *bitches* carried 62 days, one 61, and one 58; which gives, in four animals only, a latitude of four days.

Twenty-five *sows* littered, from 109 to 133 days.

Between the extremes of gestation of 161 *rabbits*, there was an interval of eight days: one of these was the 27th day, the other the 35th.

*Incubation.*—Several turkeys having sat on the eggs of *hens*, the duration of the sitting was from 17 to 27 days; the same on *ducks'* eggs, 27 days; the same on *turkeys'* eggs, from 26 to 29 days.

Hens sat on *ducks'* eggs, from 26 to 34 days; on those of their own species, from 19 to 24 days.

In *ducks*, the duration of sitting was from 28 to 32 days.

*Common geese*, from 29 to 33 days.

*Pigeons*, from 17 to 20 days.

### *Medical Statistics.*

*Extracts from the Bills of Mortality, drawn by the twelve Municipalities of Paris, for the Year* 1818.

| | | |
|---|---|---|
| The number of deaths in the city during the year, was | | 21,821 |
| of which, there happened in private residences | | 14,478 |
| Of the male sex | 7,183 | |
| — female | 7,295 | |
| the remaining | | 7,343 |

took place in hospitals; of which, 3,633 were males, and 3,710 females.

The number of persons who died from small-pox, was ... 682

It is well known, that this afflicting result from the small-pox, the ravages of which have lately been progressive, can only be attributed to the influence of the priests, who preach against vaccination.

Amongst the 21,821 deaths, are comprised 257 which occurred in jails, or of persons whose dead bodies were deposited in the offices of the police; of which there were 202 males and 55 females.

Those from suicide have not been distinctly calculated, but the number appears to have been very considerable; and it has, for several years past, progressively increased.

The principal causes of mortality are stated as follows:

| | Men. | Women. | Total. |
|---|---|---|---|
| Fevers, putrid or adynamic | 400 | 443 | 843 |
| ——— malignant or ataxic | 391 | 424 | 715 |
| ——— intermittents | 171 | 319 | 490 |
| Phlegmasiæ, cutaneous | 746 | 649 | 1,395 |
| ———— of the mucous membranes | 1,237 | 1,453 | 2,690 |
| ———— of the serous membranes | 202 | 281 | 483 |
| ———— of the cellular tissue and the parenchymatous organs | 1,454 | 1,858 | 3,312 |
| Comatous affections | 496 | 503 | 972 |
| Spasmodic ——— | 787 | 732 | 1,519 |
| Local nervous | 501 | 512 | 1,013 |
| Organic lesions, general | 1,895 | 2,063 | 3,958 |
| ————, particular | 802 | 900 | 1,702 |
| Grangrenous inflammation | 80 | 101 | 181 |
| Puerperal women | | 75 | |

Many children died from spasmodic affection, at an early age; for instance:

| | |
|---|---|
| From one day to three months ...... | 250 |
| — three months to six months... | 126 |
| — six months to one year ...... | 232 |
| — one year to two years ...... | 341 |
| — two years to three years .... | 117 |

| | Males. | Females. | Total. |
|---|---|---|---|
| There died before the age of three months | 2,202 | 1,752 | 3,944 |
| Before the age of two years ........... | 3,434 | 3,033 | 6,467 |

The total number of deaths between two and five years,

| | |
|---|---|
| was .................................... | 1,784 |
| Between five and ten............ | 1,034 |
| — ten and twenty ........ | 1,257 |
| — twenty and thirty ...... | 1,658 |
| — thirty and forty ........ | 1,480 |
| — forty and fifty .......... | 1,568 |
| — fifty and sixty .......... | 1,786 |
| — sixty and seventy ...... | 2,281 |
| — seventy and eighty ..... | 2,006 |
| — eighty and ninety ...... | 781 |
| — ninety and ninety-five... | 59 |
| — ninety-five and a hundred | 7 |
| Above a hundred ............. | 0 |

We have drawn up this last account in the same order with that of our own Bills, that the reader may compare them without trouble.

It is observed, that an important reform is requisite in the constituting of the Bills of Paris, and that the "conseil de salubrité," attached to the "prefecture de police," is engaged in the perfecting of them. It is very evident, that a similar measure respecting our own is very desirable.

---

Amongst the list of *Prize Questions* in the Number of our Journal for April last, we stated, that the question respecting *Uterine Hæmorrhage* was again proposed, by the "Société Medicale" of Paris, for the ensuing year; the commission not considering that either of the Memoirs presented possessed that degree of excellency that they desired to be evinced on so important a subject, although honourable mention was made of two, especially, and medals of reward conferred on their authors. It will be interesting to many of our readers, to hear that Madame Boivin received the second; and we feel much pleasure in thus signalizing the merits of that learned and very intelligent gentlewoman. She has recently published a translation, with notes of considerable merit and value, of the Treatises of Drs. Rigby and Stewart on the same subject. Her whole acquirements indeed shew, that nothing more than a proper mode of professional education is requisite, in order to render her sex fully competent to the practice of the obstetric part of the medical art. We wish our limits would permit us to indulge in the transcription of the

Report made on the Memoir to which we have alluded; but we can only observe, that it appears she had not effected all that was expected, only because she had indulged too far in anatomical and physiological observations, which occupied so large a proportion of her Essay, that "on pourrait presque dire que, plus tard, le temps lui a manqué pour deployer sur les points qui ont le plus de rapports avec l'objet du concours, cette richesse de connaissances pratiques qu'on aime à trouver dans les productions de ce genre." With respect to the preliminary part it is observed, that they are "claires, methodique, et bien dévelopées;" and on the pathological, "il brille surtout par un esprit d'ordre et une méthode qui méritent de grandes éloges." Our readers, who have visited the *Hospice de la Maternité*, will read the above extracts with much gratification, and utter a vow for such an establishment in London.

---

*Observations on Phosphorescence in Terrestrial Animals, (continued from page 453.)*—The *lampyris*, known by the name of the *glow-worm*, bears the phosphorescent matter at the posterior extremity of the abdomen, about the two or three last rings: they can, as well as the former species of elater, voluntarily conceal this luminous substance. When it shines most vividly, it evolves a bluish, or emerald-green, light; when it is very feeble, it displays merely a slight orange-coloured glow. This light is excited by the motion of the insects. The yellowish luminous substance may be removed from the animal, and it continues *phosphorescent* as long as it remains soft, whether in the open air or in the vacuum of the pneumatic apparatus. It loses this light on becoming dry, but it may be recovered on moistening the substance with warm water. Cold water extinguishes the light of glow-worms, but they evolve it in hot water. These insects live for a long period in a vacuum and in diverse non-respirable gases; but they all perish in the sulphurous, nitrous, and hydrochloric. Some persons have thought that hydrogen gas was sometimes rendered more detonating by their stay in it for some time.

By means of this light, the sexes meet each other: the females are the most brilliant, for the purpose of attracting the males; for the former do not often develop wings. Geer has observed, that the nymphs were also luminous. They are very common in hot climates; and, when flying about, they appear like sparks of fire amongst the flowers and shrubs, producing a very agreeable effect in the evening during summer. Carradori and Lichtenberg have remarked, that the light of these insects becomes more bright when they are plunged in oxygen gas. There are several species of luminous lampyris: the *lampyris noctiluca* and *splendida* are our glow-worms; the *italica* is the luciola of the Italians; there are also the *ignita*, *phosphorea*, *nitidula*, *lucida*, *Japonica*, *Pensylvanica*, &c. Gueneau of Montbeliard has observed, that these insects lose all their phosphorescence after copulation.

Aszelius discovered another coleoptere, the illumination of which is very singular. Its two antennæ are dilated at their extremity into little globes, and these globes are two phosphorescent lanterns, with which it enlightens the night. This insect, the *paussus spherocerus*, is described in the *Linnæan Transactions*, tome iv.

Amongst the hemiptereal insects, the *fulgores* present the phenomenon of phosphorescence in a remarkable degree. Mlle. Merian has described the great lantern-bearer, (*fulgora lanternaria*, L.) of Surinam and South-America: its front is developed into an enormous spherical vesicle, which is full of a luminous matter so bright, that one may by its means read the smallest characters in the darkest night. It appears, that this lucidity is not present during the whole life of the insect; but it is, without doubt, at the epoch of their sexual passions, as in the lampyres. There are many others of this species equally luminous; as the *fulgora candelaria*, and F. *pyrorhynchus* of Asia. Grasshoppers are also phosphorescent, according to Olivier.

Patrick Browne has also witnessed a species of nocturnal butterfly, *pyralis minor*, which presents on the inferior part of the abdomen a feeble and vacillating light, becoming obscure at intervals. It is probable that many gnats and moths, the males of which so often fly into the flame of our lamps, recognize their females during the night, only by means of feeble lights which our eyes do not discern; for all nocturnal insects that come and throw themselves into the flame, instead of flying from it, well evince (as the lampyres, &c.) that they find each other by means of their luminous appearance.

The scolopendræ have, under some circumstances, been seen to display a lucid brightness, either electric or phosphoric, according to Geer and other observers: such is the *scolopendra electrica* of Europe, and the *scolopendra phosphorea*, seen in Asia by Ekeberg. These venomous insects also avoid the open day.

It may then be established, that the phosphorescence of insects depends on an organization peculiar to many species of nocturnal animals; that it enables them to discern each other during the epoch of their sexual passions; and that the gelatinous matter, whence it is derived, only evinces this lucidity during the time of the greatest vigour of the animals, since it is extinct even before their death, when they have engendered. Since a chemical analysis has not been made of this luminous matter, and only some experiments on it in different gases effected by Grotthus, Carradori, Spallanzani, &c. we cannot conclude that its lucidity depends on phosphorus. We always find phosphates of lime and of magnesia in analyses of many insects.

Earth-worms or lumbrici, on some occasions, and when they leave their habitations for the purpose of copulation, have appeared phosphorescent. (See Flaugergues, *sur le Phosphorisme des Vers de Terre;* Journal de Physique, tome xvi. p. 311; and J. G. Bruguiere, *Journal d'Histoire Naturelle*, tome ii. p. 267.) We are ignorant of the cause of this phenomenon, which, however, is principally witnessed during the generative epochs of the animals. (Virey, *Journal de Pharmacie*, Jan. 1819.)

The first part of a French translation of Dr. Wilson Philip's Treatise on Febrile Diseases, by Dr. Letie, was published in Paris last month.

### MONTHLY CATALOGUE OF MEDICAL BOOKS.

### REPORT OF DISEASES.

THE month just elapsed, anticipated with so much anxiety by the habitual invalid, and the course of which usually shews the physician the great importance of vigilant observation of disease, and its connexion with certain states of the atmosphere, has passed away without having been the medium of such extensive destruction of human life as is usually witnessed in our climate at this period of the year; a result that may probably be attributed to the mild and regular temperature of the atmosphere with which it has been accompanied.

The frequent variation and extensive range of the temperature and general character of our climate, is usually a subject of lamentation to the valetudinarian; and it is common also to see the physician join him in his expressions of complaint: but, are there not benefits dependant on those phenomena, as well as the evils which we attribute to them? Does not each season produce in the animal economy a certain order of actions, and on passing away leave impressions more or less strongly marked, in proportion to its power and the length of time that its influence has existed? And, if other seasons did not in their turn replace them, would not those impressions become in a certain degree ineffaceable, and their results almost habitual; the consequences of which would be a sort of automatical development of the physical functions of the animal economy, that would necessarily be accompanied with an analogous want of activity and variety in the intellectual faculties? And is not such a state apparent in the inhabitants of those parts of the earth, where there is an almost constant uniformity in the state of the atmosphere and in the qualities of the soil?

Typhous fever continues to be prevalent amongst the lower class of the inhabitants, and in the more closely-peopled districts of the metropolis; the observations we made respecting its character in our last Report, will also apply to its present form. We may say the same with respect to the greater part of the reigning maladies.

Measles and whooping-cough have been more common. The former disease has been in general of a mild character, and accompanied with but little pulmonary affection. Whooping-cough is no longer the destructive disease that it was a few years since, when notions were entertained by the vulgar that it would run a certain course, in spite of the exertions of medical aid.

Various affections, of an inflammatory character, of the more superficial parts of the body are now common, especially in the applicants to public Infirmaries, &c.: the most interesting are diseases of the cutaneous organ, and inflammation of the external tunics of the eye. A free use of agents on the general economy, and but little dependance on topical measures, mark the conduct of the greater proportion of medical practitioners in the treatment of this disease.

We may here observe, that a common subject of conversation amongst the surgeons of the metropolis, is that of Syphilis; and the opinion generally expressed is, that the disease described under this term by Hunter has become very rare. Mercury is not often now employed in the treatment of "*venereal diseases*," even by those surgeons who would not hesitate in having recourse to its use for the cure of *chancre*. Gonorrhœa is of frequent occurrence; but there are few surgeons whose opinions merit attention, that do not now consider this affection to depend on different causes from that productive of the former malady.

### METEOROLOGICAL JOURNAL,

**By Messrs. WILLIAM HARRIS and Co. 50, Holborn, London.**

***From the 20th April to the 19th May, 1819, inclusive.***

| Day of Month. | Moon. | Rain gauge | Therm. | | | Barom. | | De Luc's Hygrom. Dry | Damp | | Wind. | | Atmospheric Variation. | | |
|---|---|---|---|---|---|---|---|---|---|---|---|---|---|---|---|
| Apr. | | | | | | | | | | | | | | | |
| 20 | | ,02 | 54 | 59 | 49 | 29·73 | 29·71 | | 12 | 11 | W | SW | Fog | Rain | Clo. |
| 21 | | ,03 | 53 | 57 | 45 | 29·67 | 29·77 | | 11 | 10 | WSW | NW | Clo. | | Fine |
| 22 | | | 49 | 52 | 43 | 29·88 | 29·75 | | 9 | 10 | NE | NE | Clo. | | Fine |
| 23 | | | 47 | 50 | 43 | 29·63 | 29·57 | | 10 | 10 | ENE | SE | Clo. | | |
| 24 | ● | | 45 | 48 | 44 | 29·60 | 29·64 | | 11 | 11 | E | E | Rain | | |
| 25 | | ,15 | 45 | 45 | 38 | 29 80 | 30·02 | | 11 | 11 | ESE | ENE | Rain | | Fine |
| 26 | | ,18 | 42 | 49 | 39 | 30·12 | 30·15 | | 9 | 8 | ENE | E | Fine | | |
| 27 | | | 45 | 49 | 39 | 30·18 | 30·20 | | 6 | 7 | ESE | E | Fine | | |
| 28 | | ,04 | 44 | 50 | 43 | 30·12 | 30·14 | | 9 | 6 | SE | E | Fine | | |
| 29 | | | 48 | 51 | 41 | 30·07 | 30·00 | | 5 | 5 | SE | E | Fine | | |
| 30 | | ,03 | 47 | 50 | 43 | 29·95 | 29·90 | | 2 | 3 | SSE | SSW | Fog | Fine | |
| May | | | | | | | | | | | | | | | |
| 1 | | | 45 | 57 | 45 | 29·89 | 29·85 | | 3 | 2 | S | WSW | Fine | | |
| 2 | ☽ | | 50 | 61 | 51 | 29·75 | 29.65 | | 2 | 2 | SE | SSE | Fine | | Sho. |
| 3 | | ,15 | 57 | 65 | 56 | 29·62 | 29·58 | | 3 | 5 | ESE | SE | Fine | | Clo. |
| 4 | | ,05 | 57 | 62 | 51 | 29·53 | 29·54 | | 8 | 12 | SSE | ESE | Rain | Clo. | Rain |
| 5 | | ,88 | 55 | 62 | 50 | 29·61 | 29·80 | | 10 | 10 | S | WSW | Clo. | Fine | |
| 6 | | | 57 | 63 | 48 | 29·96 | 29·53 | | 8 | 8 | SW | SSW | Clo. | | Fine |
| 7 | | | 53 | 64 | 53 | 30·06 | 30·06 | | 8 | 8 | SE | SE | Clo. | | |
| 8 | | | 58 | 63 | 55 | 30·03 | 30·08 | | 9 | 9 | ESE | S | Clo. | Fine | |
| 9 | ○ | | 59 | 70 | 53 | 30·10 | 30·14 | | 7 | 8 | W | W | Fine | | Rain |
| 10 | | ,12 | 57 | 69 | 57 | 30·07 | 30·16 | | 8 | 9 | NW | W | Fine | | Clo. |
| 11 | | | 59 | 66 | 57 | 30·16 | 30·13 | | 9 | 9 | W | WNW | Clo. | | |
| 12 | | | 62 | 65 | 55 | 30·10 | 30 09 | | 9 | 9 | WNW | NW | Clo. | | |
| 13 | | | 60 | 66 | 49 | 30·06 | 30·14 | | 9 | 8 | NW | NW | Fine | | |
| 14 | | | 55 | 64 | 49 | 30·15 | 30·11 | | 9 | 8 | NNW | N | Fine | | |
| 15 | | | 54 | 61 | 49 | 30·06 | 30·04 | | 7 | 8 | NE | SSE | Fine | Clo. | |
| 16 | ☾ | | 55 | 64 | 48 | 30·02 | 30·00 | | 9 | 9 | SSE | S | Fine | Clo. | |
| 17 | | | 55 | 67 | 52 | 29·96 | 29·88 | | 9 | 5 | SE | S | Fine | | |
| 18 | | | 57 | 67 | 50 | 29·83 | 29·87 | | 6 | 7 | WSW | WSW | Fine | | |
| 19 | | ,15 | 55 | 62 | 49 | 29·63 | 29·60 | | 9 | 13 | NE | S | Rain | | |

The quantity of rain fallen in the month of April, is 2 inches and 13-100ths.

